# 2-Meth­oxy­quinoline-3-carbaldehyde

**DOI:** 10.1107/S1600536810034744

**Published:** 2010-09-04

**Authors:** K. Chandraprakash, P. Ramesh, K. Ravichandran, P. S. Mohan, M. N. Ponnuswamy

**Affiliations:** a Department of Chemistry, School of Chemical Sciences, Bharathiar University, Coimbatore 641 046, India; b Centre of Advanced Study in Crystallography and Biophysics, University of Madras, Guindy Campus, Chennai 600 025, India

## Abstract

In the title compound, C_11_H_9_NO_2_, the quinoline ring system is essentially planar (r.m.s. deviation = 0.005 Å) and the meth­oxy and aldehyde groups are almost coplanar with it [N—C—O—C = 6.24 (19) and O—C—C—C = 0.3 (2)°]. In the crystal, mol­ecules are linked by pairs of C—H⋯O hydrogen bonds, forming centrosymmetric *R*_2_^2^(10) dimers. The dimers are linked *via* π–π inter­actions involving the pyridine and benzene rings [centroid–centroid distance = 3.639 (1) Å].

## Related literature

For general background to quinoline derivatives, see: Mali *et al.* (2010 ▶); Kuethe *et al.* (2003 ▶). For hydrogen-bond motifs, see: Bernstein *et al.* (1995 ▶).
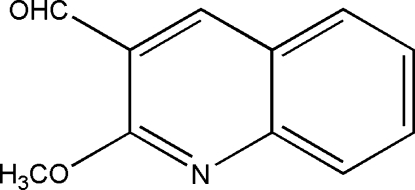


## Experimental

### 

#### Crystal data

C_11_H_9_NO_2_
*M* *_r_* = 187.19
Monoclinic, 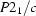

*a* = 8.8206 (6) Å
*b* = 4.8446 (3) Å
*c* = 21.6828 (14) Å
β = 90.612 (4)°
*V* = 926.50 (10) Å^3^
*Z* = 4
Mo *K*α radiation
μ = 0.09 mm^−1^
*T* = 293 K
0.20 × 0.20 × 0.18 mm

#### Data collection

Bruker SMART APEXII area-detector diffractometer
Absorption correction: multi-scan (*SADABS*; Bruker, 2008 ▶) *T*_min_ = 0.981, *T*_max_ = 0.983
8812 measured reflections
2305 independent reflections
1658 reflections with *I* > 2σ(*I*)
*R*_int_ = 0.030

#### Refinement

*R*[*F* ^2^ > 2σ(*F* ^2^)] = 0.044
*wR*(*F* ^2^) = 0.130
*S* = 1.05
2305 reflections
128 parameters
H-atom parameters constrained
Δρ_max_ = 0.16 e Å^−3^
Δρ_min_ = −0.19 e Å^−3^

### 

Data collection: *APEX2* (Bruker, 2008 ▶); cell refinement: *SAINT* (Bruker, 2008 ▶); data reduction: *SAINT*; program(s) used to solve structure: *SHELXS97* (Sheldrick, 2008 ▶); program(s) used to refine structure: *SHELXL97* (Sheldrick, 2008 ▶); molecular graphics: *ORTEP-3* (Farrugia, 1997 ▶); software used to prepare material for publication: *SHELXL97* and *PLATON* (Spek, 2009 ▶).

## Supplementary Material

Crystal structure: contains datablocks global, I. DOI: 10.1107/S1600536810034744/ci5162sup1.cif

Structure factors: contains datablocks I. DOI: 10.1107/S1600536810034744/ci5162Isup2.hkl

Additional supplementary materials:  crystallographic information; 3D view; checkCIF report

## Figures and Tables

**Table 1:** Hydrogen-bond geometry (Å, °)

*D*—H⋯*A*	*D*—H	H⋯*A*	*D*⋯*A*	*D*—H⋯*A*
C4—H4⋯O2^i^	0.93	2.56	3.4157 (16)	152

Symmetry code: (i) 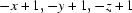.

**Fig. 1.**
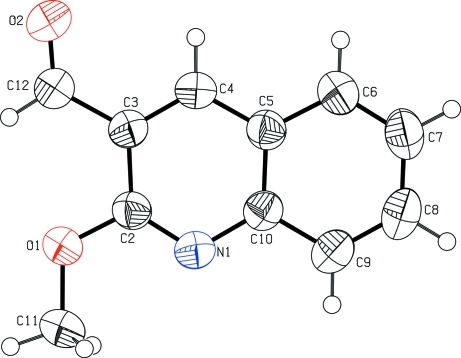
The molecular structure of the title compound, showing the atomic numbering and displacement ellipsoids drawn at the 50% probability level.

**Fig. 2.**
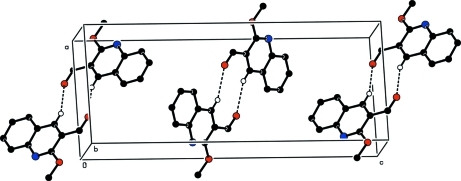
The crystal packing of the title compound. H atoms not involved in hydrogen bonding (dashed lines) have been omitted for clarity.

## supplementary crystallographic information

### Comment

Quinolines have gained importance in medicinal and natural product chemistry due to their interesting biological and pharmacological activities. They possess anti-malarial, anti-tuberculosis, anti-inflammatory and anti-cancer properties (Mali *et al.*, 2010). Methoxy substituted quinolines are used as synthetic intermediates in the construction of novel class of KDR kinase inhibitors (Kuethe *et al.*, 2003). Against this background and to ascertain the structure of title compound, the crystallographic studies have been carried out.

In the title molecule (Fig.1), the quinoline ring system (N1/C2–C10) is essentially planar with a maximum deviation of 0.007 (1) Å for atom C3. The methoxy and carbaldehyde groups are almost coplanar with the quinoline ring system, which is evidenced from torsion angles C3—C2—O1—C11 and C2—C3—C12—O2 of 173.6 (1)° and 178.5 (2)°, respectively.

The packing of the molecules in the crystal is stabilized by C—H···O, and π–π types of intermolecular interactions. The molecules at (*x*, *y*, *z*) and (1-*x*, 1-*y*, 1-*z*) are linked by a pair of intermolecular C4—H4···O2 hydrogen bonds to form a centrosymmetric dimer containing *R*_2_^2^(10) ring motif (Fig. 2) (Bernstein *et al.*, 1995). The π–π interaction between the pyridine ring (N1/C2-C10) of the quinoline ring system at (x, y, z) and the benzene ring (C5—C10) at (x, y-1, z) further stabilize the structure, with a centroid-centroid distance of 3.639 (1) Å.

### Experimental

To a solution of 1 g (17.8 mmol) of KOH in 50 ml of MeOH was added 2.5 g (13.1 mmol) of 2-chloro-3-quinolinecarboxaldehyde. The mixture was heated at 373 K for 2.5 h and then cooled to room temperature, and poured into 200 g of crushed ice. The precipitate thus obtained was recuperated by filtration. The obtained product was a colourless solid. The product was purified by recrystallization from petroleum ether-ethyl acetate mixture.

### Refinement

H atoms were positioned geometrically (C–H = 0.93–0.96 Å) and allowed to ride on their parent atoms, with *U*_iso_(H) = 1.5*U*_eq_(C) for methyl H and 1.2*U*_eq_(C) for other H atoms.

### Crystal data

**Table:**

C_11_H_9_NO_2_	*F*(000) = 392
*M**_r_* = 187.19	*D*_x_ = 1.342 Mg m^−^^3^
Monoclinic, *P*2_1_/*c*	Mo *K*α radiation, λ = 0.71073 Å
Hall symbol: -P 2ybc	Cell parameters from 1216 reflections
*a* = 8.8206 (6) Å	θ = 1.9–28.4°
*b* = 4.8446 (3) Å	µ = 0.09 mm^−^^1^
*c* = 21.6828 (14) Å	*T* = 293 K
β = 90.612 (4)°	Block, colourless
*V* = 926.50 (10) Å^3^	0.20 × 0.20 × 0.18 mm
*Z* = 4	

### Data collection

**Table:**

Bruker SMART APEXII area-detector diffractometer	2305 independent reflections
Radiation source: fine-focus sealed tube	1658 reflections with *I* > 2σ(*I*)
graphite	*R*_int_ = 0.030
ω and φ scans	θ_max_ = 28.4°, θ_min_ = 1.9°
Absorption correction: multi-scan (*SADABS*; Bruker, 2008)	*h* = −9→11
*T*_min_ = 0.981, *T*_max_ = 0.983	*k* = −6→6
8812 measured reflections	*l* = −27→28

### Refinement

**Table:**

Refinement on *F*^2^	Primary atom site location: structure-invariant direct methods
Least-squares matrix: full	Secondary atom site location: difference Fourier map
*R*[*F*^2^ > 2σ(*F*^2^)] = 0.044	Hydrogen site location: inferred from neighbouring sites
*wR*(*F*^2^) = 0.130	H-atom parameters constrained
*S* = 1.05	*w* = 1/[σ^2^(*F*_o_^2^) + (0.0622*P*)^2^ + 0.1045*P*] where *P* = (*F*_o_^2^ + 2*F*_c_^2^)/3
2305 reflections	(Δ/σ)_max_ = 0.001
128 parameters	Δρ_max_ = 0.16 e Å^−^^3^
0 restraints	Δρ_min_ = −0.19 e Å^−^^3^

### Special details

**Table:**

Geometry. All e.s.d.'s (except the e.s.d. in the dihedral angle between two l.s. planes) are estimated using the full covariance matrix. The cell e.s.d.'s are taken into account individually in the estimation of e.s.d.'s in distances, angles and torsion angles; correlations between e.s.d.'s in cell parameters are only used when they are defined by crystal symmetry. An approximate (isotropic) treatment of cell e.s.d.'s is used for estimating e.s.d.'s involving l.s. planes.
Refinement. Refinement of *F*^2^ against ALL reflections. The weighted *R*-factor *wR* and goodness of fit *S* are based on *F*^2^, conventional *R*-factors *R* are based on *F*, with *F* set to zero for negative *F*^2^. The threshold expression of *F*^2^ > σ(*F*^2^) is used only for calculating *R*-factors(gt) *etc*. and is not relevant to the choice of reflections for refinement. *R*-factors based on *F*^2^ are statistically about twice as large as those based on *F*, and *R*- factors based on ALL data will be even larger.

### Fractional atomic coordinates and isotropic or equivalent isotropic displacement parameters (Å^2^)

**Table:**

	*x*	*y*	*z*	*U*_iso_*/*U*_eq_	
O1	−0.04660 (11)	0.2962 (2)	0.42027 (4)	0.0664 (3)	
O2	0.32252 (12)	0.2311 (3)	0.52502 (5)	0.0860 (4)	
N1	0.05048 (11)	0.6258 (2)	0.35559 (5)	0.0522 (3)	
C2	0.06704 (13)	0.4656 (3)	0.40308 (5)	0.0498 (3)	
C3	0.20052 (14)	0.4490 (3)	0.44096 (5)	0.0505 (3)	
C4	0.31810 (14)	0.6144 (3)	0.42593 (6)	0.0546 (3)	
H4	0.4064	0.6107	0.4497	0.066*	
C5	0.30810 (14)	0.7921 (3)	0.37465 (6)	0.0502 (3)	
C6	0.42741 (16)	0.9652 (3)	0.35643 (7)	0.0639 (4)	
H6	0.5179	0.9658	0.3789	0.077*	
C7	0.41177 (18)	1.1312 (3)	0.30644 (7)	0.0682 (4)	
H7	0.4913	1.2450	0.2947	0.082*	
C8	0.27587 (18)	1.1311 (3)	0.27260 (6)	0.0656 (4)	
H8	0.2658	1.2462	0.2385	0.079*	
C9	0.15831 (16)	0.9657 (3)	0.28878 (6)	0.0594 (3)	
H9	0.0691	0.9678	0.2655	0.071*	
C10	0.17083 (14)	0.7915 (2)	0.34040 (5)	0.0476 (3)	
C11	−0.17739 (17)	0.2814 (4)	0.38058 (7)	0.0822 (5)	
H11A	−0.2189	0.4630	0.3750	0.123*	
H11B	−0.2521	0.1638	0.3989	0.123*	
H11C	−0.1487	0.2076	0.3413	0.123*	
C12	0.21226 (17)	0.2551 (3)	0.49311 (6)	0.0634 (4)	
H12	0.1288	0.1449	0.5017	0.076*	

### Atomic displacement parameters (Å^2^)

**Table:**

	*U*^11^	*U*^22^	*U*^33^	*U*^12^	*U*^13^	*U*^23^
O1	0.0566 (5)	0.0821 (7)	0.0603 (6)	−0.0219 (5)	−0.0105 (4)	0.0149 (5)
O2	0.0747 (7)	0.1034 (9)	0.0792 (7)	−0.0159 (6)	−0.0249 (6)	0.0365 (7)
N1	0.0530 (6)	0.0557 (6)	0.0476 (5)	−0.0036 (5)	−0.0056 (4)	0.0005 (5)
C2	0.0503 (7)	0.0530 (7)	0.0460 (6)	−0.0067 (5)	−0.0017 (5)	−0.0021 (5)
C3	0.0527 (7)	0.0535 (7)	0.0453 (6)	−0.0035 (5)	−0.0034 (5)	0.0014 (5)
C4	0.0504 (7)	0.0603 (8)	0.0529 (7)	−0.0056 (6)	−0.0084 (5)	0.0023 (6)
C5	0.0532 (7)	0.0488 (7)	0.0486 (6)	−0.0042 (5)	0.0001 (5)	−0.0024 (5)
C6	0.0608 (8)	0.0660 (9)	0.0648 (8)	−0.0129 (7)	−0.0002 (6)	0.0044 (7)
C7	0.0759 (9)	0.0609 (8)	0.0679 (9)	−0.0156 (7)	0.0118 (7)	0.0043 (7)
C8	0.0894 (10)	0.0541 (8)	0.0536 (7)	0.0015 (7)	0.0092 (7)	0.0081 (6)
C9	0.0694 (8)	0.0574 (8)	0.0512 (7)	0.0043 (6)	−0.0030 (6)	0.0039 (6)
C10	0.0555 (7)	0.0441 (6)	0.0432 (6)	0.0010 (5)	0.0002 (5)	−0.0039 (5)
C11	0.0647 (9)	0.1064 (13)	0.0750 (10)	−0.0338 (9)	−0.0203 (8)	0.0167 (9)
C12	0.0607 (8)	0.0698 (9)	0.0595 (8)	−0.0115 (7)	−0.0078 (6)	0.0136 (7)

### Geometric parameters (Å, °)

**Table:**

O1—C2	1.3510 (15)	C6—C7	1.356 (2)
O1—C11	1.4336 (16)	C6—H6	0.93
O2—C12	1.1932 (16)	C7—C8	1.399 (2)
N1—C2	1.2964 (16)	C7—H7	0.93
N1—C10	1.3736 (16)	C8—C9	1.360 (2)
C2—C3	1.4307 (16)	C8—H8	0.93
C3—C4	1.3534 (17)	C9—C10	1.4050 (18)
C3—C12	1.4728 (18)	C9—H9	0.93
C4—C5	1.4081 (18)	C11—H11A	0.96
C4—H4	0.93	C11—H11B	0.96
C5—C6	1.4055 (18)	C11—H11C	0.96
C5—C10	1.4136 (17)	C12—H12	0.93
			
C2—O1—C11	117.35 (10)	C8—C7—H7	120.0
C2—N1—C10	117.35 (10)	C9—C8—C7	121.09 (13)
N1—C2—O1	120.34 (10)	C9—C8—H8	119.5
N1—C2—C3	125.09 (11)	C7—C8—H8	119.5
O1—C2—C3	114.57 (11)	C8—C9—C10	120.39 (13)
C4—C3—C2	117.15 (11)	C8—C9—H9	119.8
C4—C3—C12	120.99 (12)	C10—C9—H9	119.8
C2—C3—C12	121.83 (11)	N1—C10—C9	119.18 (12)
C3—C4—C5	120.70 (11)	N1—C10—C5	122.35 (11)
C3—C4—H4	119.7	C9—C10—C5	118.47 (12)
C5—C4—H4	119.7	O1—C11—H11A	109.5
C6—C5—C4	123.08 (12)	O1—C11—H11B	109.5
C6—C5—C10	119.56 (12)	H11A—C11—H11B	109.5
C4—C5—C10	117.36 (11)	O1—C11—H11C	109.5
C7—C6—C5	120.57 (13)	H11A—C11—H11C	109.5
C7—C6—H6	119.7	H11B—C11—H11C	109.5
C5—C6—H6	119.7	O2—C12—C3	123.89 (13)
C6—C7—C8	119.91 (13)	O2—C12—H12	118.1
C6—C7—H7	120.0	C3—C12—H12	118.1
			
C10—N1—C2—O1	−179.57 (11)	C5—C6—C7—C8	0.1 (2)
C10—N1—C2—C3	0.28 (19)	C6—C7—C8—C9	−0.3 (2)
C11—O1—C2—N1	6.24 (19)	C7—C8—C9—C10	0.4 (2)
C11—O1—C2—C3	−173.63 (13)	C2—N1—C10—C9	179.19 (11)
N1—C2—C3—C4	0.2 (2)	C2—N1—C10—C5	−0.34 (18)
O1—C2—C3—C4	−179.94 (11)	C8—C9—C10—N1	−179.80 (12)
N1—C2—C3—C12	−178.08 (13)	C8—C9—C10—C5	−0.25 (19)
O1—C2—C3—C12	1.78 (18)	C6—C5—C10—N1	179.55 (12)
C2—C3—C4—C5	−0.62 (19)	C4—C5—C10—N1	−0.06 (18)
C12—C3—C4—C5	177.68 (12)	C6—C5—C10—C9	0.02 (18)
C3—C4—C5—C6	−179.05 (13)	C4—C5—C10—C9	−179.60 (11)
C3—C4—C5—C10	0.56 (19)	C4—C3—C12—O2	0.3 (2)
C4—C5—C6—C7	179.67 (14)	C2—C3—C12—O2	178.47 (15)
C10—C5—C6—C7	0.1 (2)		

### Hydrogen-bond geometry (Å, °)

**Table:**

*D*—H···*A*	*D*—H	H···*A*	*D*···*A*	*D*—H···*A*
C4—H4···O2^i^	0.93	2.56	3.4157 (16)	152

Symmetry codes: (i) −*x*+1, −*y*+1, −*z*+1.
